# Role of Electronegativity in Environmentally Persistent Free Radicals (EPFRs) Formation on ZnO

**DOI:** 10.1021/acs.jpcc.3c08231

**Published:** 2024-03-15

**Authors:** Syed Monjur Ahmed, Reuben A. Oumnov, Orhan Kizilkaya, Randall W. Hall, Phillip T. Sprunger, Robert L. Cook

**Affiliations:** 1 Department of Chemistry, Louisiana State University, Baton Rouge, Louisiana 70803, United States; 2 Department of Natural Sciences and Mathematics, Dominican University of California, San Rafael, California 94901, United States; 3 Center for Advanced Microstructures and Devices, Louisiana State University, 6980 Jefferson Highway, Baton Rouge, Louisiana 70806, United States; 4 Department of Physics and Astronomy, Louisiana State University, Baton Rouge, Louisiana 70803, United States

## Abstract

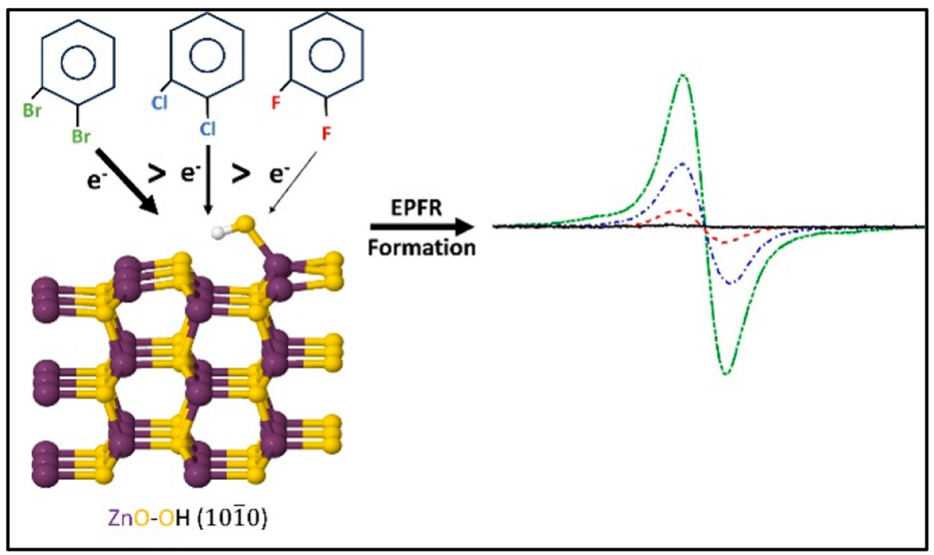

Environmentally persistent free radicals (EPFRs), a group of emerging pollutants, have significantly longer lifetimes than typical free radicals. EPFRs form by the adsorption of organic precursors on a transition metal oxide (TMO) surface involving electron charge transfer between the organic and TMO. In this paper, dihalogenated benzenes were incorporated to study the role of electronegativity in the electron transfer process to obtain a fundamental knowledge of EPFR formation mechanism on ZnO. Upon chemisorption on ZnO nanoparticles at 250 °C, electron paramagnetic resonance (EPR) confirms the formation of oxygen adjacent carbon-centered organic free radicals with concentrations between 10^16^ and 10^17^ spins/g. The radical concentrations show a trend of 1,2-dibromobenzene (DBB) > 1,2-dichlorobenzene (DCB) > 1,2-difluorobenzene (DFB) illustrating the role of electronegativity on the amount of radical formation. X-ray absorption spectroscopy (XAS) confirms the reduction of the Zn^2+^ metal center, contrasting previous experimental evidence of an oxidative mechanism for ZnO single crystal EPFR formation. The extent of Zn reduction for the different organics (DBB > DCB > DFB) also correlates to their polarity. DFT calculations provide theoretical evidence of ZnO surface reduction and exhibit a similar trend of degree of reduction for different organics, further building on the experimental findings. The lifetimes of the EPFRs formed confirm a noteworthy persistency.

## 1 Introduction

A free radical is generally an atom or molecule with an unpaired electron and is unstable (i.e., short lifetime) in nature.^1^ Environmentally persistent free radicals (EPFRs) are organic free radicals stabilized on or inside particles and exist in significant concentrations in contaminated superfund soils,^2^ clays,^3^ and atmospheric particulate matter (PM).^4−6^ Combustion systems and thermal processes such as waste incineration, automobile combustion engines, refineries, metal smelting process, and biomass burning generate an abundant quantity of ultrafine particles, aromatic hydrocarbons, and transition metal oxides (TMOs), which subsequently produce EPFRs in the cooling zone of a waste incinerator or smelter.^7−9^ Studies have shown that particles released from traffic, coal combustion, cigarette smoke, and dust carry large amounts of EPFRs.^10−13^ Airborne PM has been shown to cause significant negative health impacts.^14−16^ In addition to the size, shape, and composition of PM, the organic molecules associated with PM also determine the toxicity of PM.^14^ PM that contains EPFRs/TMO systems can damage heart and lung as EPFRs can form reactive oxygen species (ROS) that sequentially can cause oxidative stress and cell death in both humans and animals.^17−20^ EPFRs adversely affect human health, causing cardiovascular and respiratory dysfunction,^21^ asthma,^22^ reduced blood supplies,^23^ and influenza infection,^24^ and have a negative effect on the environment.^25^

**Figure 1:**
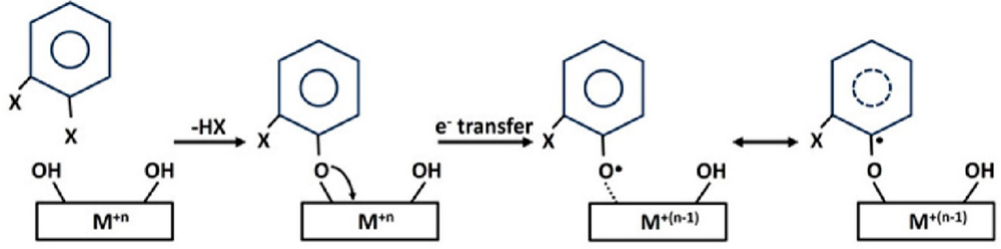
Schematic of EPFR formation from an organic precursor adsorbed on a generic metal oxide surface.

EPFRs have been found to be formed on TMO nanoparticles (NPs) by the adsorption of organic aromatic precursors at elevated temperature (375–775 K).^26^ Various aromatic and substituted aromatic precursors such as phenol, 2-chlorophenol, hydroquinone, catechol, and 1,2-dichlorobenzene have been studied for the formation of EPFRs on the surface of different transition metal oxides, such as CuO, Al_2_O_3_, Fe_2_O_3_, TiO_2_, and ZnO.^27,28^ The proposed mechanism of EPFR formation involves initial physisorption followed by thermally activated chemisorption via removal of a small molecule (HX), depending on the kind of the organic precursor, and, at last, a redox process.^8^ Generally, a single or partial electron charge is transferred between the organic precursor and the transition metal oxide after chemisorption, resulting in EPFR formation,^8^ as illustrated in Figure 1 for a metal reduction mechanism.

EPFRs formed by the association of an organic precursor and a transition metal oxide generally include semiquinone, phenoxyl, and cyclopentadienyl type radicals.^29^ For example, phenol and substituted phenols form phenoxyl type radicals, and hydroquinone and substituted hydroquinones form semiquinone type radicals.^8^ It has been found that catechol, 2-chlorophenol, and 1,2-dichlorobenzene can form both phenoxyl and semiquinone type radicals.^8^ The radicals thus formed can produce either oxygen-centered or carbon-centered EPFRs (see Figure 1).^30,31^ EPFRs are resonance-stabilized radicals,^32^ and therefore resist degradation, and persist in ambient environment.^33^ EPFRs have much longer half-lives than most typical free radicals and can persist for hours, weeks, or months.^34^ The longevity of an EPFR depends on both the type of organic precursor and the type of transition metal oxide.^35^

Studies have shown that the size of supported TMO NPs governs the generation and yield of EPFRs.^36^ Xu et al. have found that the catalytic ability of a metal oxide to form EPFRs increases with decreasing particle size.^37^ As nanoparticle sized TMOs have higher relative surface areas (surface-to-bulk ratio), and therefore more active sites,^37^ it is anticipated that nanosized TMO particles will generate higher EPFR concentrations than the same quantity of larger TMO particles. Moreover, it is reported that EPFRs can be subjected to thermal activation above a certain temperature (typically >220 °C), resulting in the increased spin density of the organic radical formed.^38,39^

As EPFR formation involves electron charge transfer between an organic precursor and a metal redox center,^8^ a question arises as to the role of electronegativity, χ, in EPFR formation and characteristics. In terms of the organic precursor, the effect of substituents and electronegativity, pivotal for EPFR formation and attributes, has yet to be studied. Numerous substituted organics are ubiquitous in various industrial processes and household products,^40,41^ and a comparative study of substituents regarding polarity will provide a better understanding of the EPFR formation mechanism on a fundamental level. A starting point for such an investigation can be a study of halogenated, such as brominated (χ(Br) = 2.8), chlorinated (χ(Cl) = 3.0), and fluorinated (χ(F) = 4.0), benzenes. In this article, dihalogenated benzenes, namely, 1,2-dibromobenzene (DBB), 1,2-dichlorobenzene (DCB), and 1,2-difluorobenzene (DFB), were incorporated to acquire novel insights into the role of electronegativity on the EPFR formation mechanism. A ZnO nanoparticle system was chosen as the transition metal oxide of interest due to the fact that ZnO has been found to oxidize readily during EPFR formation mechanism,^42,43^ contrasting the proposed default metal oxide reduction mechanism of EPFR formation.^27^ Moreover, ZnO and dihalogenated benzene systems were chosen as a bottom-up approach for the fundamental study of EPFRs formation. A more complex system with combined different metal oxides and organics to mimic the real-world system can be subjected to future study. Electron paramagnetic resonance (EPR) spectroscopy was used to detect and quantify formed radicals as well as to study the persistency of the radicals. X-ray absorption near edge structure (XANES) spectroscopy technique was utilized to investigate the oxidation state change of the Zn metal center. Theoretical modeling and calculations were combined in this work to corroborate the experimental data.

## 2 Experimental Methods

### 2.1 Chemicals and Materials

ZnO nanopowder (18 nm, stock no. US3599, CAS no. 1314-13-2, 99.95% purity, SSA 40–70 m^2^/g) was purchased from US Research Nanomaterials. 1,2-Dibromobenzene (catalog no. 225000-25G, CAS no. 583-53-9, density 1.956 g/mL, 98% purity) was purchased from Beantown Chemical (BTC). 1,2-Dichlorobenzene (CAS no. 95-50-1, density 1.3 g/mL, 98+% purity) was purchased from Acros Organics. 1,2-Difluorobenzene (CAS no. 367-11-3, density 1.17 g/mL, >98.0% purity) was purchased from Tokyo Chemical Industry (TCI). All the chemicals and materials were used as received and without any further purification.

### 2.2 Gas Phase Dosing of ZnO Nanoparticle

The samples were prepared in a custom-made vacuum dosing manifold mentioned in a previous work.^43^ Briefly, the nanopowder (100 mg) and the organics were combined (1:1 w/w ratio for complete surface saturation) in a detachable 10 mm quartz sample EPR tube. The EPR tube was connected to the dosing manifold via a detachable glass arm containing a valve to seal the vacuum. Originally, the sample tube was opened to the pumps to remove oxygen and other contaminants. This was done to ensure that the reaction happened only between the metal oxide and the dihalogenated organic. The sample tube was closed to the pump after the attainment of desired pressure (∼10^–4^ Torr). Thereafter, the sample was heated to 250 °C (to mimic combustion process) and allowed to react for 1 h. After 1 h, the sample tube was again opened to the pumps for 1 h at the dosing temperature to ensure that unreacted dihalogenated organics were removed and thus cannot interfere with subsequent EPR analysis. Finally, after the sample was cooled to room temperature, and the valve of the glass arm was closed to keep the sample under vacuum and taken for measurements. The final pressure inside the sample tube was ∼10^–4^ Torr.

### 2.3 EPR Analysis

EPR measurements were performed using a Bruker EMX X-band dual cavity spectrometer, a modulation frequency of 100 kHz, and a microwave frequency of ∼9.7 GHz. The measurements were taken at room temperature, and the operating parameters were the following: microwave power ∼2.0 mW, modulation amplitude 4 G, sweep width 250 G, time constant 0.640 ms, sweep time 41.943 s, and number of scans 3. Samples were prepared in triplicate to ensure data accuracy and reproducibility. Radical concentrations were calculated by comparing the signal peak area to that of a 2,2-diphenyl-1-picrylhydrazyl (DPPH) standard of a known amount.

### 2.4 X-ray Absorption Spectroscopy Study

X-ray absorption near edge structure (XANES) Zn K-edge measurements were obtained at the wavelength shifter double-crystal monochromator (WDCM) beamline of J. Bennett Johnston, Sr., Center for Advanced Microstructures and Devices (CAMD), Louisiana State University, Baton Rouge, Louisiana. The monochromator has a channel cut Si-111 crystal.. Zinc metal foil was used for monochromator calibration at 9659 eV. Samples were prepared by smearing the powder onto a Kapton tape and folding the tape a few folds to increase the sample thickness. The spectra were obtained in transmission mode at room temperature, and multiple scans (2–4 scans) were performed for each sample for data accuracy and a better signal-to-noise ratio. The data was analyzed using Athena software where the spectra were aligned, merged, and truncated.

X-ray absorption near edge structure (XANES) Zn L-edge spectra were acquired at the variable-line-spaced plane grating monochromator (VLSPGM) beamline of the CAMD synchrotron facility. The samples were spread onto a carbon tape placed on a stainless-steel sample holder and loaded into the sample chamber maintained at ∼10^–9^ Torr pressure via load-lock chamber. All measurements were attained at room temperature in total electron yield mode, and multiple scans (3–4 scans) were taken for each sample for data accuracy and reproducibility. ZnO powder was used as a reference for the energy calibration. The spectra were normalized and analyzed by using Athena software.

### 2.5 Theoretical Modeling

The ZnO crystal structure of Kihara and Donnay^44^ was used to generate a 3 × 3 × 2 and a 3 × 3 × 3 nonpolar (1010) surface using the CrystalMaker X software.^45^ The orthorhombic crystal lattice constants were *a* = 3.2493 Å and *c* = 5.2040 Å. The cell length was 30 Å in the direction perpendicular to the surface. *Ab initio* density functional calculations using the generalized gradient approximation (GGA) were performed using the Quantum Espresso software package.^46,47^ The GGA functionals of Perdew-Bruke-Ernzerhof (PBE) are given along with local density approximation (LDA). Pseudopotentials came from the pslibrary provided by Quantum Espresso (QE). The plane wave cutoffs for the energy and charge density were 71 and 496 Ry, respectively. Gaussian smearing was used with a width of 0.1 Ry. DFT+U calculations were used with *U* = 5.2 eV as suggested by Maldonado et al.^48^ A vacuum dipole correction using a sawtooth potential, as contained in QE, used eamp = 0.0, emaxpos = 0.67, and eopreg = 0.05. The QE-supplied Grimme-D3 semiempirical van der Waals correction was used. The Γ point was used for the calculations. Density of states were calculated using QE and cube files created by QE were used to perform Bader charge analysis on the charge and spin densities.^49−52^

### 2.6 Lifetime Study

To determine the stability and persistency of the EPFRs formed by ZnO dosed with dihalogenated benzenes, kinetic studies were performed. The samples were stored at room temperature in dark and under vacuum, and the EPR signal was measured periodically to determine the radical concentration as a function of time. A first-order kinetic equation was used to calculate reaction rate,^53^ −d*R*/d*t* = *k*[*R*], where *R* is the concentration of formed EPFRs. The 1/e lifetime (*t*_1/e_) of the EPFRs was calculated by the following first-order decay expression:^53^ ln(*R*/*R*_o_) = −*kt* and *t*_1/e_ = 1/*k*. Here, *k* is the rate constant derived from the slope of the correlation between the natural logarithm of relative radical concentration (*R/R*_o_) versus time (*t*), and 1/e lifetime (*t*_1/e_) was deduced thereby. Samples were prepared and characterized in triplicates to verify data accuracy and reproducibility.

## 3 Results and Discussion

### 3.1 EPR Study

**Figure 2:**
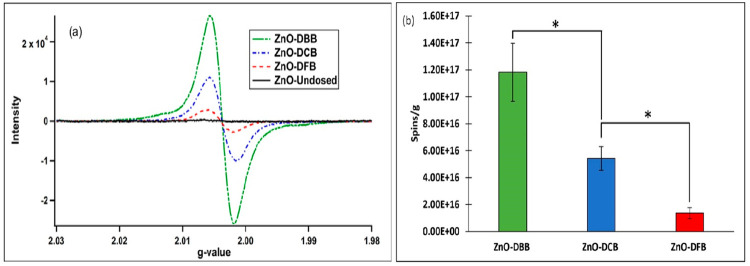
(a) Average EPR spectra. (b) Average concentration of formed EPFRs in spins/g. The undosed ZnO did not form any EPFR. Error bars indicate standard deviation (SD). * indicates *p* < 0.0005.

The EPR results show the formation and characteristics of organic radicals by ZnO nanoparticles dosed with DBB, DCB, and DFB (Figure 2). The amount of radicals formed decreased monotonically from DBB to DFB with the average radical concentrations of (11.80 ± 0.21) × 10^16^ spins/g, (5.42 ± 0.89) × 10^16^ spins/g, and (1.35 ± 0.41) × 10^16^ spins/g for DBB, DCB, and DFB, respectively. The benzene derivative with the lowest electronegative halogen bromine [χ(Br) = 2.8] yields the highest amount of EPFRs, and with the highest electronegative halogen fluorine [χ(F) = 4.0] it produces the lowest amount of EPFRs, with chlorine [χ(Cl) = 3.0] being in-between.

Bromine will have the lowest electron affinity and fluorine will have the highest due to their electronegativity; hence, it will be electrostatically easiest for the electrons to transfer from the organic precursor to the ZnO metal surface with DBB and hardest with DFB, thus producing the highest amount of radicals for DBB by facilitating the metal center reduction process, and the least amount for DFB. The undosed ZnO sample does not form any radicals or EPR signal (solid black line in Figure 2a) and provides direct evidence that the obtained EPR signals are due to the organics on the ZnO surface. The spectral characteristics are summarized in Table S1 in the Supporting Information. The average g-values range from 2.0035 to 2.0039 and indicative of carbon-centered radical with an adjacent oxygen atom.^29^ All of the samples show narrow EPR peak width (Δ*H*_p-p_) with average peak-to-peak distances of 5.89–7.33 G.

### 3.2 XAS Study

**Figure 3:**
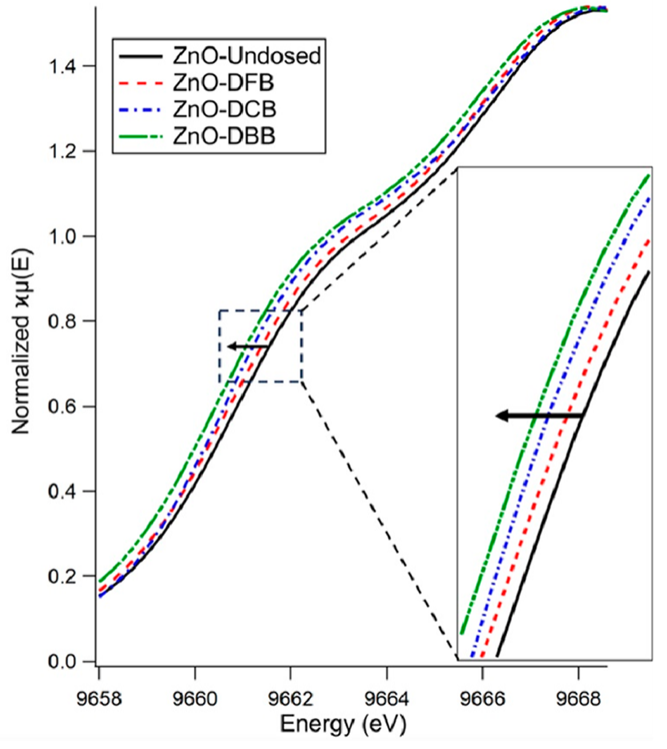
Zn K-edge XANES normalized average spectra of ZnO-Undosed (black), ZnO-DFB (red), ZnO-DCB (blue), and ZnO-DBB (green line).

To better elucidate the redox mechanism of metal–organic coupling during EPFR formation and the corresponding pattern of EPR results, DBB-, DCB-, and DFB-dosed ZnO samples were analyzed by X-ray absorption near edge structure (XANES) spectroscopy. Figure 3 shows the Zn K-edge absorption spectra of undosed and dosed ZnO nanoparticles. Figure S6 in the Supporting Information contains the entire spectra. The edge position, obtained from the first maximum of the derivative curve of the Zn K edge spectra, for the dosed samples shifts toward lower photon energy compared to the undosed sample from 9660.82 ± 0.02 eV for undosed-ZnO to 9660.62 ± 0.04 eV, 9660.51 ± 0.03 eV, and 9660.43 ± 0.02 eV for DFB-, DCB-, and DBB-dosed ZnO, respectively. Zn K-edge absorption line arises from the 1s → 4p electronic transition,^54^ and a shift in K-edge toward lower photon energy reflects a reduction process due to decreased oxidation state (increased electron density) of the metal center resulting in lower binding energy of the electrons.^3^

The edge-shifts of the average spectra are 0.20, 0.31, and 0.39 eV for ZnO-DFB, ZnO-DCB, and ZnO-DBB, respectively, with respect to the edge of the undosed ZnO XANES spectrum. The extent of the shifts (DBB > DCB > DFB) for the dosed samples follows an inverse trend, with the electronegativity of the halogens (F > Cl > Br) on the aromatic precursors. This trend supports the EPR results and is consistent with Zn metal reduction, as fluorine is the most electronegative element among the halogens; it will have the strongest electrostatic attraction on the electrons and vice versa for bromine, with chlorine in-between. Thus, the polarity of the organics affects the electron transfer potential during EPFR formation, resulting in varied extent of Zn metal center reduction of ZnO.

**Figure 4:**
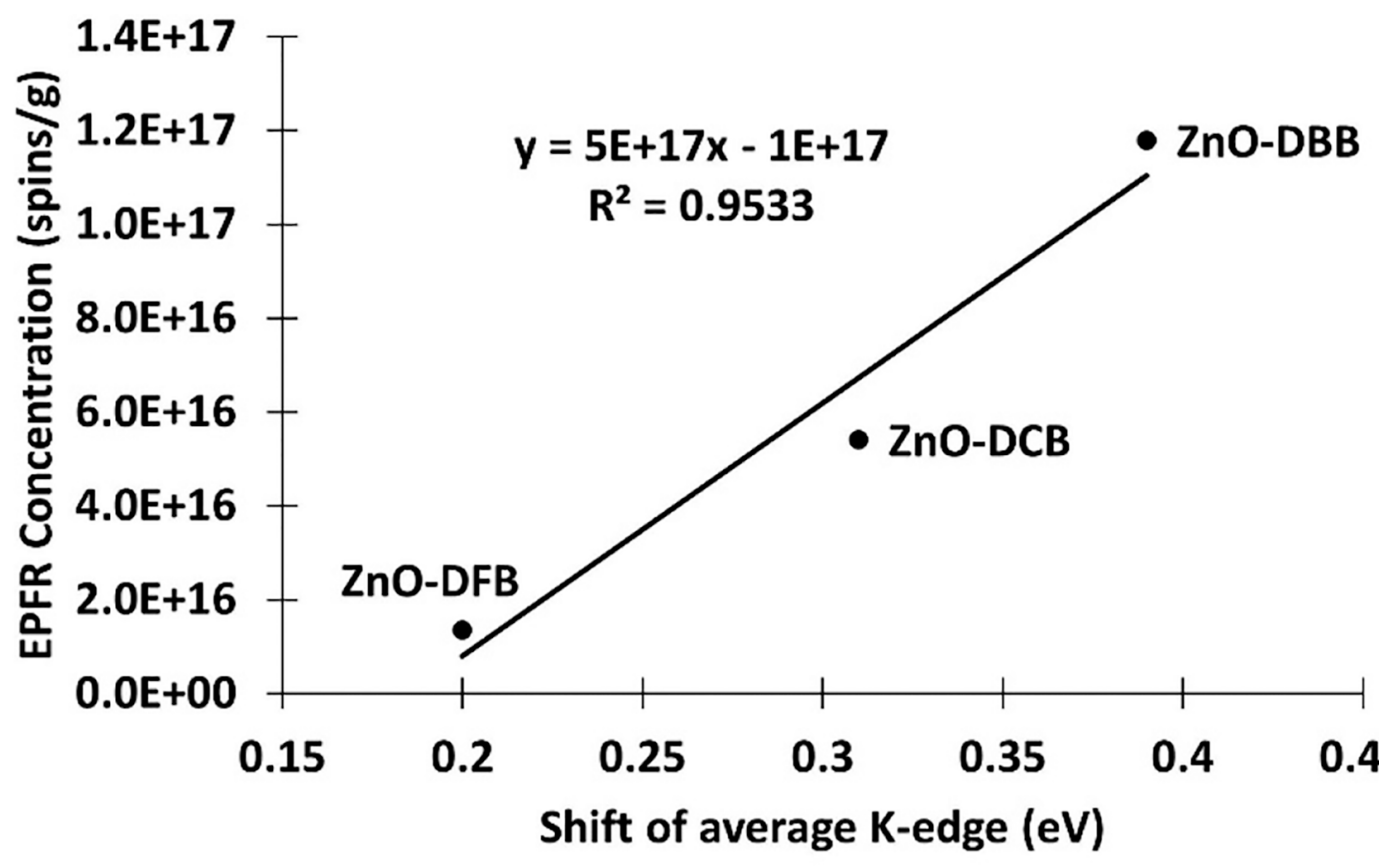
Proportional relation between the shift of the average K-edge of the dosed ZnO samples with respect to the undosed ZnO and the formed EPFR concentration.

The quantity of the formed radicals exhibits a linear proportionality with the redox potential of the metal–organic system (Figure 4). A larger shift in metal center K-edge results in higher amount of EPFR formation. This correlation demonstrates that the degree of metal center reduction caused by aromatics with different polarities influences the magnitude of radical formation. Thus, the electron charge transfer process, along with the redox potential of the organic precursor, should be considered a vital step for predicting EPFR formation. In addition, the chemical adsorption step is not a trivial but an important step for the EPFR formation process. The carbon (C)–halogen (X) bond enthalpy follows the sequence of C–F > C–Cl > C–Br, allowing the dissociation of C–Br bond to be the most energetically favorable and C–F the least.^55^ This phenomenon can also synergistically impact the radical formation shown in Figure 2. Chemisorption and electron transfer required for EPFR formation may provide an opportunity to prevent radical formation. More work is needed to fully uncover the intricacies of such processes leading to EPFR formation.

**Figure 5:**
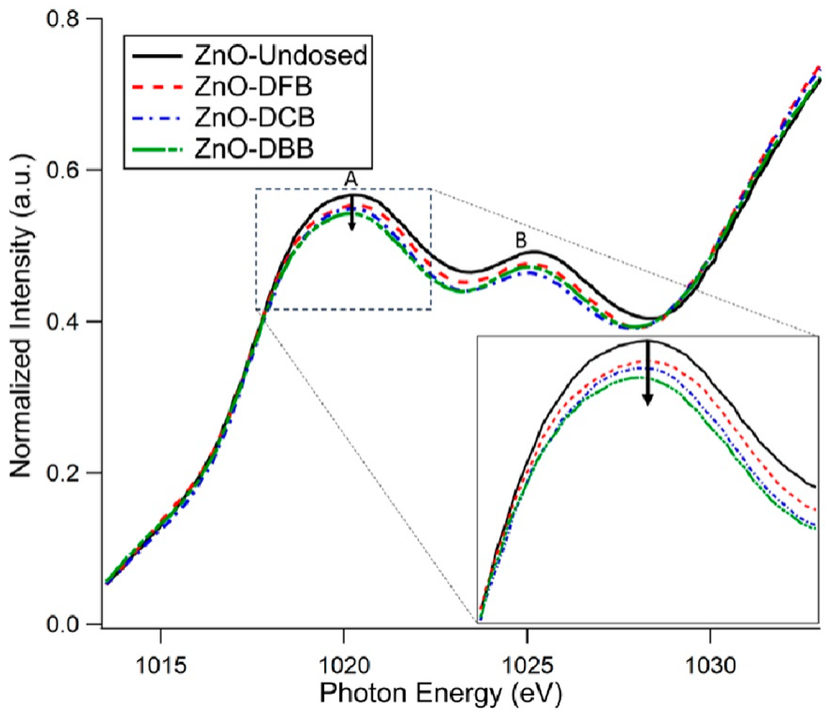
Zn L-edge XANES normalized average spectra of ZnO-Undosed (black), ZnO-DFB (red), ZnO-DCB (blue), and ZnO-DBB (green line).

To further confirm the reduction process of ZnO during EPFR formation, the L-edge of the Zn metal center of undosed and dosed ZnO nanoparticles was studied. Figure 5 shows the Zn L_3_-edge spectra of undosed ZnO and DBB-, DCB-, and DFB-dosed ZnO samples. As per the dipole-transition selection rule, Zn L_3_-edge probes the unoccupied Zn s- and d-driven electronic states.^56−58^ For ZnO, the lowest unoccupied orbital of the Zn^2+^ ion is Zn 4s, followed by 4p and 4d as the Zn 3d orbital is fully occupied.^59^ The features “A” at ∼1020 eV and “B” at ∼1025 eV of Zn L_3_-edge spectra in Figure 5 correspond to the transition of Zn 2p_3/2_ electron to the hybridized Zn 4s and predominantly 4d states, respectively.^60,61^

As the peak intensities are approximately proportional to the population of the unoccupied states,^58,62^ the change in intensity from the undosed sample to the dosed samples reveals the redox mechanism of the Zn metal center in ZnO nanoparticles. Figure 5 shows decreased intensities, i.e., less density of unoccupied states, of the dosed samples’ peaks compared to the undosed spectrum, which implies reduction of Zn metal center during EPFR formation. Moreover, ZnO-DFB has the smallest decrease in intensity while ZnO-DBB has the largest decrease with respect to the undosed ZnO. This result is consistent with the K-edge data further confirming the reduction of Zn metal center during EPFR formation on ZnO surface associated with the oxidation potential of the polar organic precursors.

A more surface sensitive technique, X-ray photoelectron spectroscopy (XPS) has also been incorporated to study the redox changes of the ZnO metal center. The spectra do not show any change at room temperature dosing, which demonstrates that the sorption of the organic precursors on ZnO without EPFR formation (before thermally activated charge transfer) does not cause any shift in spectra. Figure S7 in the Supporting Information contains the XPS spectra of ZnO sample dosed with the dihalogenated benzenes at 250 °C. The Zn peaks of the dosed samples shift toward lower binding energy with respect to the undosed sample confirming reduction of Zn metal center. The degree of shifts shows the trend of DBB > DCB > DFB which is consistent with XANES K-edge and L-edge results. The findings from XPS, XANES K-edge, and L-edge data concomitantly prove the reduction of ZnO metal center during EPFR formation and that different organics cause different extent of charge transfer due to their varied polarity induced by the varied electronegative halogens.

ZnO has been both experimentally and theoretically found to undergo oxidation process during EPFR formation with (1010) and 0001-Zn surfaces via partial charge transfer for phenol adsorbate.^42,43^ A computational study showed that ZnO nonpolar (1010) surface can be subjected to both oxidation and reduction processes depending on the adsorption site on ZnO surface.^48^ The present work is the first experimental study to demonstrate reduction of the Zn metal center during EPFR formation on the ZnO surface.

### 3.3 Theoretical Calculation

**Figure 6:**
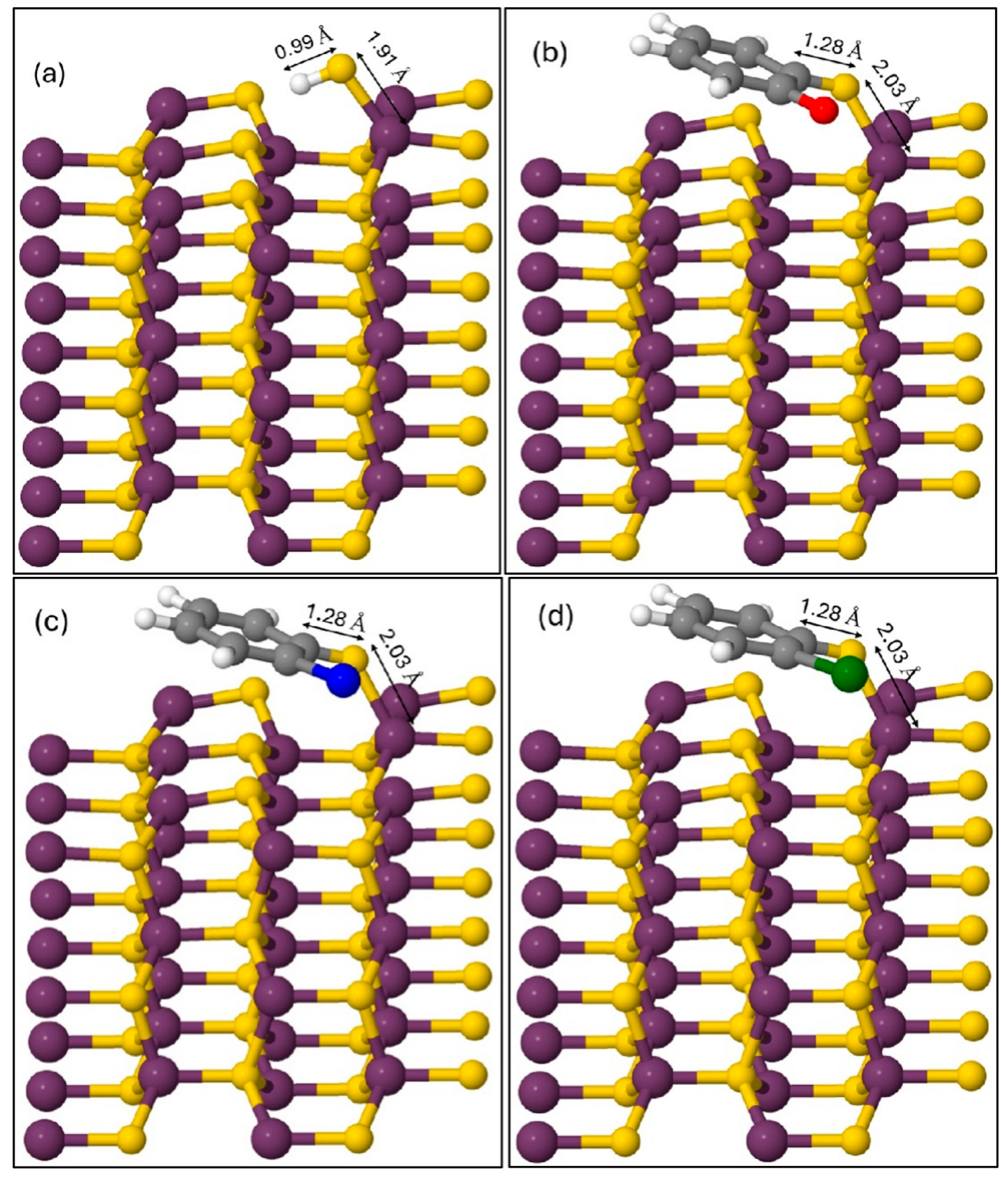
Optimized 3 × 3 × 2 ZnO nonpolar (1010) models with (a) surface hydroxyl, (b) DFB, (c) DCB, and (d) DBB attached on the surface. Zn atoms are colored purple, O atoms are colored gold, C atoms are colored gray, H atoms are colored white, F atom is colored red, Cl atom is colored blue, and Br atom is colored green. Such colors for the halogen atoms were chosen to maintain consistency with the other figures.

Theoretical models were generated, and DFT calculations were executed to provide a systemic view of the organic precursor–metal oxide conjugation system with the attempt to obtain atomistic details regarding the experimental results. The (1010) surface was chosen because it is a nonpolar surface, thus elucidating consequent charge transfer is more straightforward. For the surface adsorption of a dihalogenated benzene, one of the C–X bonds needs to be broken to form a bond with an oxygen atom (O) of a ZnO surface hydroxyl (−OH) via the removal of a small molecule HX as depicted in Figure 1.^63^Figure 6 contains the optimized final geometry models of 3 × 3 × 2 nonpolar (1010) ZnO surface with surface hydroxyl, followed by DFB, DCB, and DBB adsorbed on the surface. The halogenated benzenes coordinate with the ZnO surface via the C–O–Zn bond where the O–Zn (of C–O–Zn) bond length increases from 1.91 to 2.03 Å after the attachment of the adsorbates and the resulting C–O bond length is 1.28 Å. The subsequent increment in the remaining C–X bond length, 1.35, 1.74, and 1.91 Å for C–F, C–Cl, and C–Br, respectively, from ZnO-DFB to ZnO-DBB pertains to the size of the halogens responsible for the C–X bond energy, alongside their electronegativity value.

Bader charge analysis was performed to obtain the charge density of the four models shown in Figure 6. The results are summarized in Table 1. The bridging oxygen atom is included with the 3 × 3 × 2 ZnO surface when calculating the charge and spin of the surface as it is the part of surface hydroxyl. The calculations show that the ZnO surface has a charge of −0.15e before adsorption of the dihalogenated benzenes. The surface bound oxygen atom has a charge of −0.55e, and the remaining 3 × 3 × 2 ZnO surface has a charge of +0.40e. After the attachment of the organics, the ZnO surface became significantly more negatively charged to the value of approximately −0.92e for all three of the adsorbates. The surface bound oxygen atom has a charge of −1.1e and the remaining 3 × 3 × 2 ZnO surface has a charge of +0.20e for all three of the adsorbates. Thus, the bridging oxygen atom is reduced by −0.55e and the 3 × 3 × 2 ZnO surface is reduced by −0.2e by the addition of the adsorbates. Thus, there is a partial reduction of the ZnO surface and an oxidation of the organic due to adsorption of the aromatics as seen in the experimental data. The charge values on the halogen atoms of the organics show that they are negatively charged inducing polarity into the organic ring, which is expected as they are electronegative elements, and exhibit a trend of Br (−0.04e) < Cl (−0.20e) < F (−0.63e) which is in line with their electronegativity trend of Br (2.8) < Cl (3.0) < F (4.0). The spin density calculation (Table S2 in the Supporting Information) shows the absolute values of overall spin of the models. From Table S2, it can be seen that dominant fraction of the overall system’s spin is associated with the organic C_6_H_4_ ring, which qualitatively correlates to the experimental *g*-value.

**Table 1:** Bader Charge Density Analysis for 3 × 3 × 2 ZnO Nonpolar (1010) Models

model	ZnO surface (e)	surface hydroxyl or organic (e)	halogen (X) atom (e)
ZnO-OH	–0.15	–0.40	
ZnO-DFB	–0.92	0.92	–0.63
ZnO-DCB	–0.92	0.92	–0.20
ZnO-DBB	–0.92	0.92	–0.04

**Figure 7:**
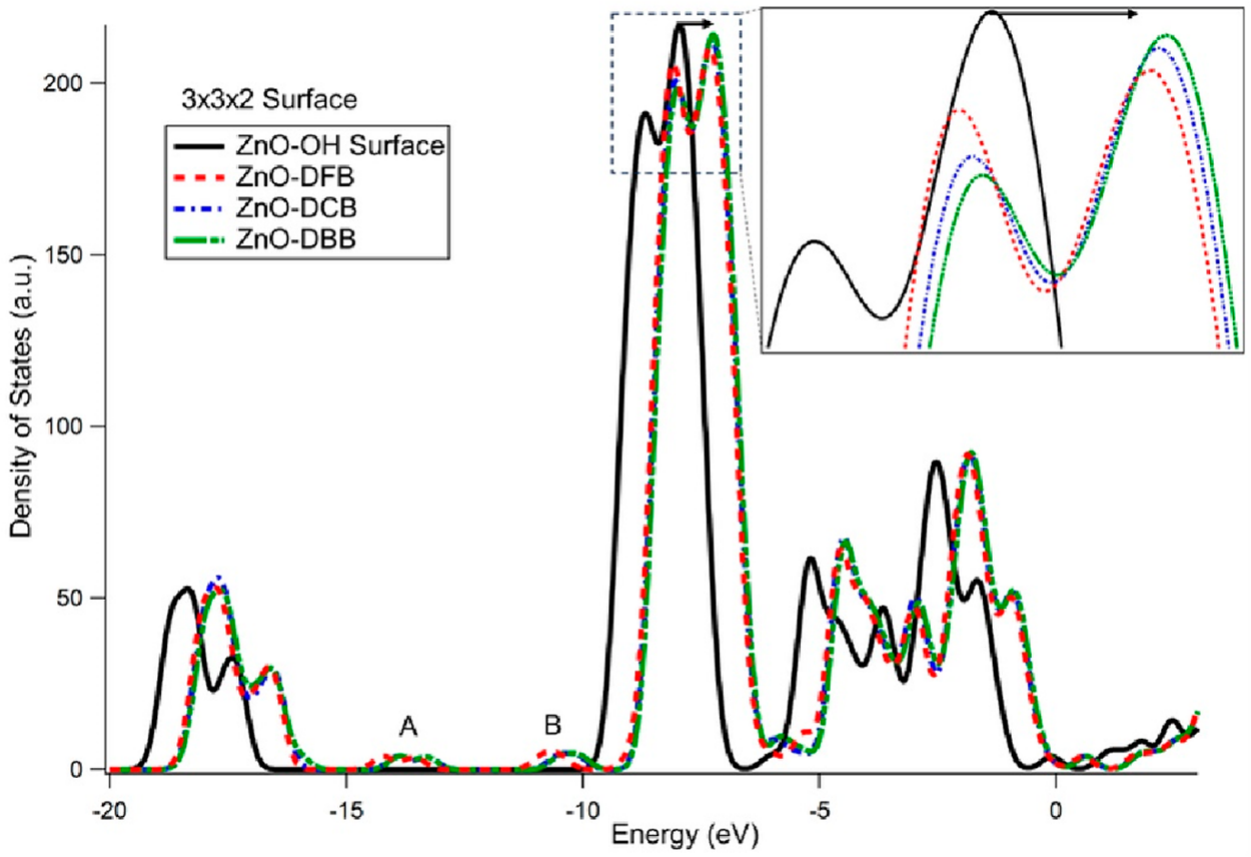
Density of state diagram of ZnO-OH (black line), ZnO-DFB (red line), ZnO-DCB (blue line), and ZnO-DBB (green line) for 3 × 3 × 2 nonpolar (1010) models.

Figure 7 reveals the density of states diagram for the aforementioned four models. Density of state is a concept about how electrons are energetically distributed.^64^ The states from −10 eV to 0 eV arise principally from the hybridized cation Zn 3d-anion O 2p states, while the states from −20 eV to −15 eV are due to O 2s/2p states.^65^ The density of state diagrams for the ZnO-DFB, ZnO-DCB, and ZnO-DBB models show the occurrence of two new features annotated as “A” and “B” between −15 eV and −10 eV range which are absent for the ZnO-OH model. These two features are due to surface-adsorbed organics, distinguishing the spectra between ZnO models with and without the dihalogenated benzenes. Moreover, the curves for the ZnO-DXB (X = F, C, B) models significantly shift toward lower binding energy compared to that of ZnO-OH model indicating reduction of ZnO surface after the attachment of aromatics which is consistent with the experimental finding from XANES K-edge and L-edge study. It is also visible from the density of states diagram that the shifts of the ZnO-DXB spectra are different, and show a trend from DFB < DCB < DBB. This pattern indicating varied extent of ZnO reduction due to the different organics with different polarity is consistent with the experimental XANES study.

ZnO nonpolar (1010) surface of 3 × 3 × 3 unit cell was also modeled, and Bader charge analysis and DFT calculations were performed (Supporting Information). The results from the 3 × 3 × 3 cell’s (Figures S1–S4) calculations are in line with that of 3 × 3 × 2 cell’s. Bader charge density analysis (Table S3) shows that the ZnO surface becomes more negatively charged after the adsorption of the organics, confirming the reduction of the ZnO surface. The analysis also shows the charges associated with the halogens of the organic precursors correspond to their electronegativity (F > Cl > Br), inducing polarity into the aromatics in consequence. The spin density calculations (Table S4) are in agreement with the 3 × 3 × 2 models and the experimental data demonstrating the spin of the radicals predominantly within the C_6_H_4_ ring. The density of states diagram for the 3 × 3 × 3 model (Figure S5) shows a shift of the valence band toward lower binding energy from the ZnO-OH model to the ZnO-DXB models, and the pattern of shifts are in line with the 3 × 3 × 2 models of DFB < DCB < DBB with respect to ZnO-OH. This study further validates the experimental and theoretical findings of the paper.

### 3.4 Lifetime Study

**Figure 8:**
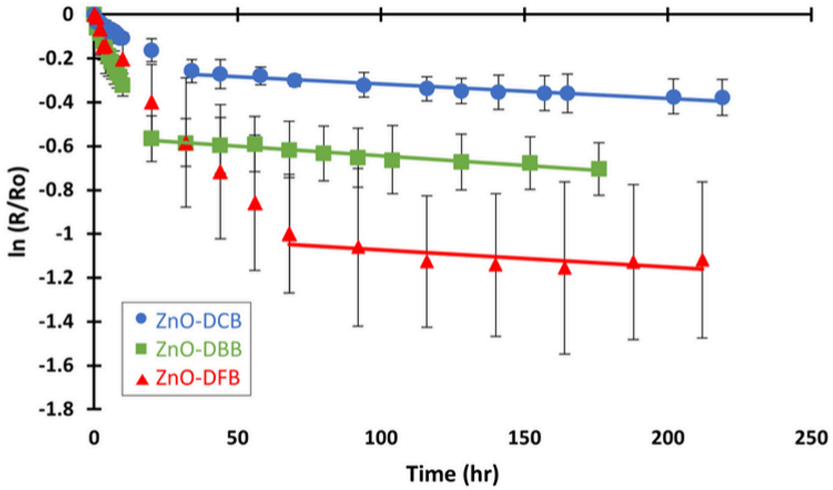
Average decay pattern of the formed EPFRs. Error bars indicate standard deviation (SD).

The formation and propagation of the EPFRs via the metal–organic complex is a delocalized resonance system, leading to the longevity of the radicals. The persistence of EPFRs contributes to their impact on human health and the environment. The lifetimes of the EPFRs formed by ZnO upon dosing with DBB, DCB, and DFB were studied to help understand their overall stability and attempt to correlate with their radical characteristics. Figure 8 shows the decay pattern of the radicals formed by the dosed samples. All of the dosed samples show similar two decay patterns: an initial fast decay, followed by slow decay. This type of decay pattern is consistent with previous studies for EPFRs.^35,66^

**Figure 9:**
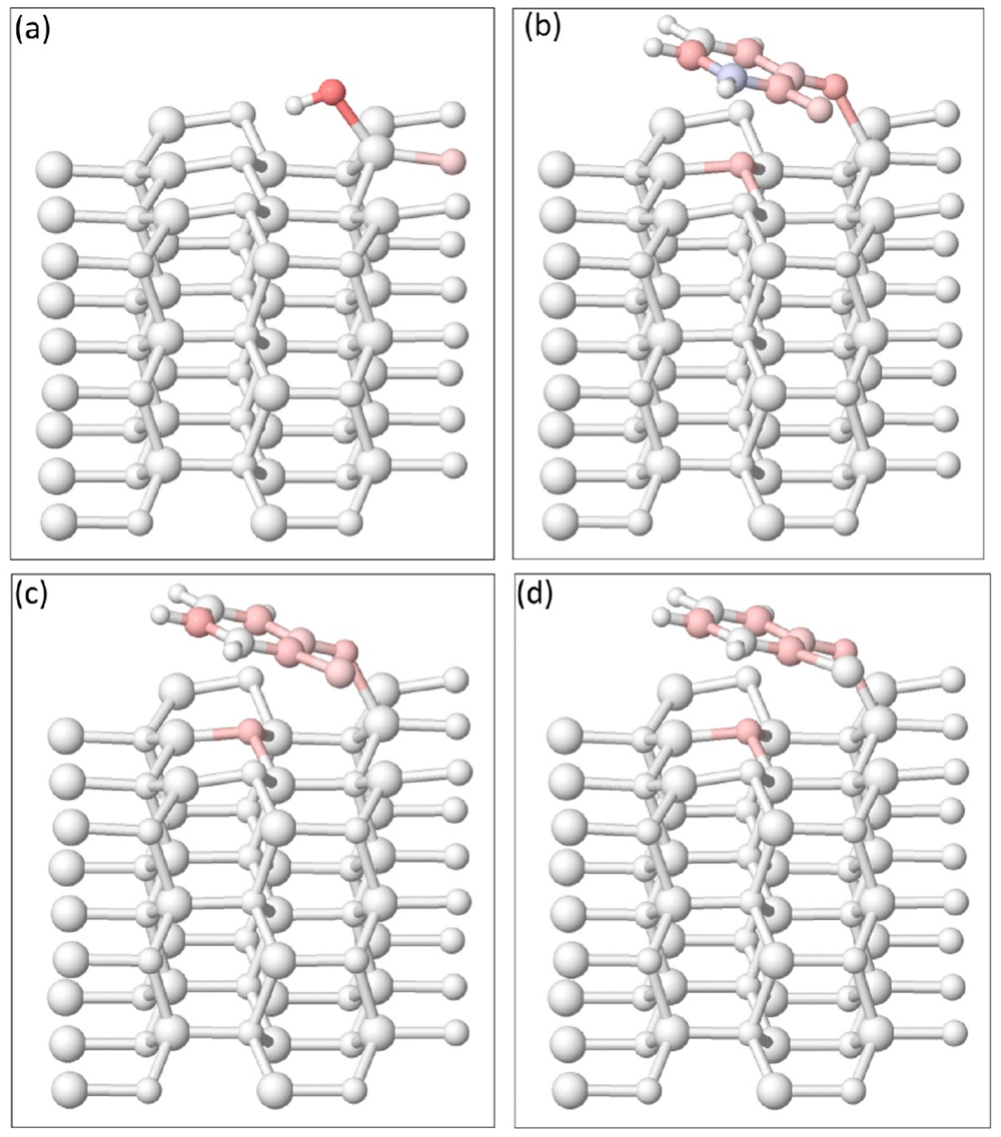
Spin density distribution of the 3 × 3 × 2 ZnO nonpolar (1010) models with (a) surface hydroxyl, (b) DFB, (c) DCB, and (d) DBB attached on the surface. Blue and red colors indicate unpaired up and down spins, respectively.

The 1/e lifetimes of the radicals are given in Table 2. EPFRs formed on ZnO surface are found to be more persistent than other transition metal oxides.^35,43^ Although the radicals in this study show initial fast decay, ultimately they exhibit similar significant stability with the average 1/e lifetime of 46.30–59.52 days for the slow decay, which is comparable to the literature with ZnO oxidation mechanism. The DFT spin density distribution (Figure 9) illustrates the delocalization of the radicals over the aromatic ring which resembles the resonance of the π structure of the organic ring, explaining the stable and relatively less reactive nature of the radicals. This lifetime study shows that EPFRs generated on the ZnO surface, regardless of the polarity of the organic precursors and metal oxide redox mechanism, demonstrate notable prolonged lifetime for which they can persist longer to impose serious health risks.

**Table 2:** Summary of the Lifetime Study of the Formed EPFRs

	ZnO-DBB	ZnO-DCB	ZnO-DFB
long lifetime ± SD (day)	46.30 ± 10.911	59.52 ± 8.906	52.08 ± 13.941

## 4 Conclusion

This work investigated the EPFRs formation mechanism on ZnO by focusing on the role of electronegativity in the electron charge transfer process between the organic precursor and the transition metal oxide (TMO). Due to the different electronegativity values of the halogens bromine [χ(Br) = 2.8], chlorine [χ(Cl) = 3.0], and fluorine [χ(F) = 4.0], dihalogenated benzenes such as 1,2-dibromobenzene (DBB), 1,2-dichlorobenzene (DCB), and 1,2-difluorobenzene (DFB) have different polarity. The EPR study confirmed the formation of organic radicals on ZnO surface after being dosed with DBB, DCB, and DFB. The amount of radical formation followed a trend of DBB > DCB > DFB, in line with the pattern of electronegativity of the halogens. This trend aligns with expectations of the organic-TMO systems’ electronegativity in a ZnO reduction process. XANES K-edge and L-edge studies support the EPR study by confirming that the Zn^2+^ metal center undergoes reduction during EPFR formation. Moreover, the magnitude of reduction, which is related to the amount of electron charge transfer, follows a trend of DBB > DCB > DFB with respect to the undosed ZnO. Theoretical calculations demonstrated the same mechanism of ZnO reduction with a similar trend of reduction for DBB > DCB > DFB, along with the different polarity and delocalization of spin density of the aromatic ring corroborating the experimental results. In concert, the findings of this study illustrated the significance of the polarity of the organic precursor in EPFR formation, revealing an alternative mechanism of EPFR formation for ZnO. Finally, the lifetime study showed that the formed EPFRs are stable enough to impose adverse effects on human health and the environment.

## Supporting Information Available

EPR spectral characteristics, spin density calculation for the 3 × 3 × 2 nonpolar (1010) models, 3 × 3 × 3 nonpolar (1010) ZnO models, Bader charge density and spin density calculations for the 3 × 3 × 3 models, the density of state diagram for the 3 × 3 × 3 models, coordinates of the optimized 3 × 3 × 2 nonpolar (1010) models’ surface, XANES K-edge full spectra, XPS spectra of the dosed and undosed ZnO samples at 250 °C (PDF)
