# Supplementary material for: Role of Electronegativity in Environmentally Persistent Free Radicals (EPFRs) Formation on ZnO
Source: J Phys Chem C Nanomater Interfaces. 2024 Mar 15;128(12):5179–88. doi: 10.1021/acs.jpcc.3c08231 (PMC10983065; doi:10.1021/acs.jpcc.3c08231)
Supplement: Supplementary file 1 — jp3c08231_si_001.pdf [file jp3c08231_si_001.pdf]

SUPPLEMENTAL INFORMATION

Role of Electronegativity in Environmentally Persistent Free Radicals (EPFRs) Formation on ZnO

Authors

Syed Monjur Ahmed[1], Reuben A. Oumnov[2], Orhan Kizilkaya[3], Randall W. Hall[2]*, Phillip T. Sprunger[3,4]*, Robert L. Cook[1]*

Affiliations

[1]Department of Chemistry, Louisiana State University, Baton Rouge, LA 70803, USA

[2]Department of Natural Sciences and Mathematics, Dominican University of California, San Rafael, CA 94901, USA

[3]Center for Advanced Microstructures and Devices, Louisiana State University, 6980 Jefferson Highway, Baton Rouge, LA 70806, USA

[4]Department of Physics and Astronomy, Louisiana State University, Baton Rouge, LA 70803, USA

Table S1. Summary of EPR Results

Sample	Average EPFR Concentration (spins/g) ± S.D.	Average g-value ± S.D.	Average ΔHp-p (G) ± S.D.
ZnO-DBB	$(11.80 \pm 0.21)\times10^{16}$	2.0037±0.00009	6.43±0.28
ZnO-DCB	$(5.42 \pm 0.89)\times10^{16}$	2.0035±0.00004	5.89±0.10
ZnO-DFB	$(1.35\pm0.41)\times10^{16}$	2.0039±0.00006	7.33±0.10

Table S2. Spin Density Analysis of 3x3x2 ZnO non-polar $(10\bar{1}0)$ models

Model	ZnO Surface	Pheno-X	C_6H_4	X	Bridging O
ZnO-DFB	-0.35	-0.54	-0.51	-0.03	-0.17
ZnO-DCB	-0.34	-0.49	-0.49	-0.03	-0.15
ZnO-DBB	-0.35	-0.44	-0.44	-0.02	-0.14
ZnO-OH	-0.48	-	-	-	-0.36

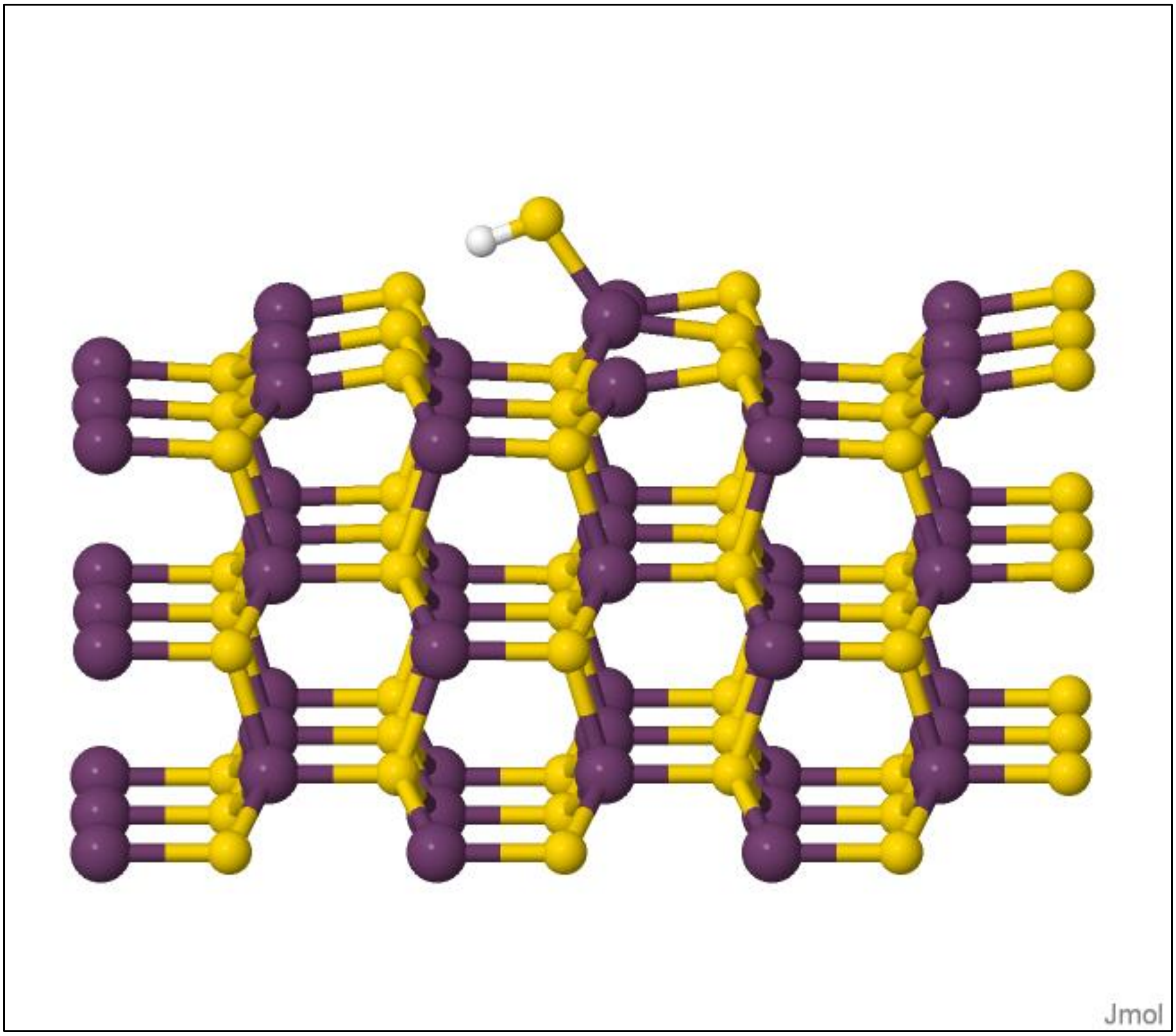

Figure S1. Optimized 3x3x3 ZnO non-polar $(10\bar{1}0)$ model with surface hydroxyl. Zn atoms are colored purple, O atoms are colored gold, and H atom is colored white.

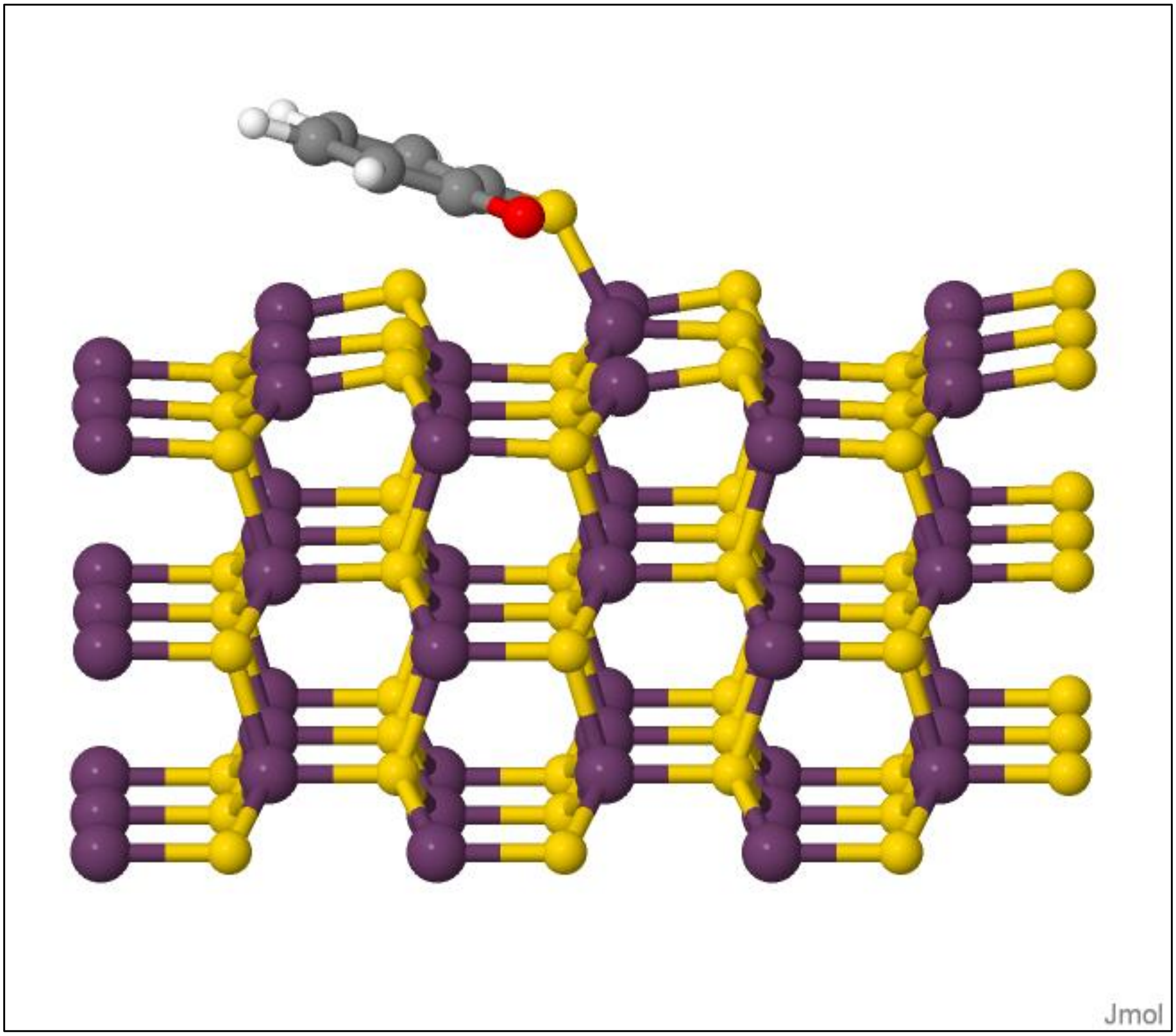

Figure S2. Optimized 3x3x3 ZnO non-polar $(10\bar{1}0)$ model with DFB attached on the surface. Zn atoms are colored purple, O atoms are colored gold, C atoms are colored gray, H atoms are colored white, and F atom is colored red.

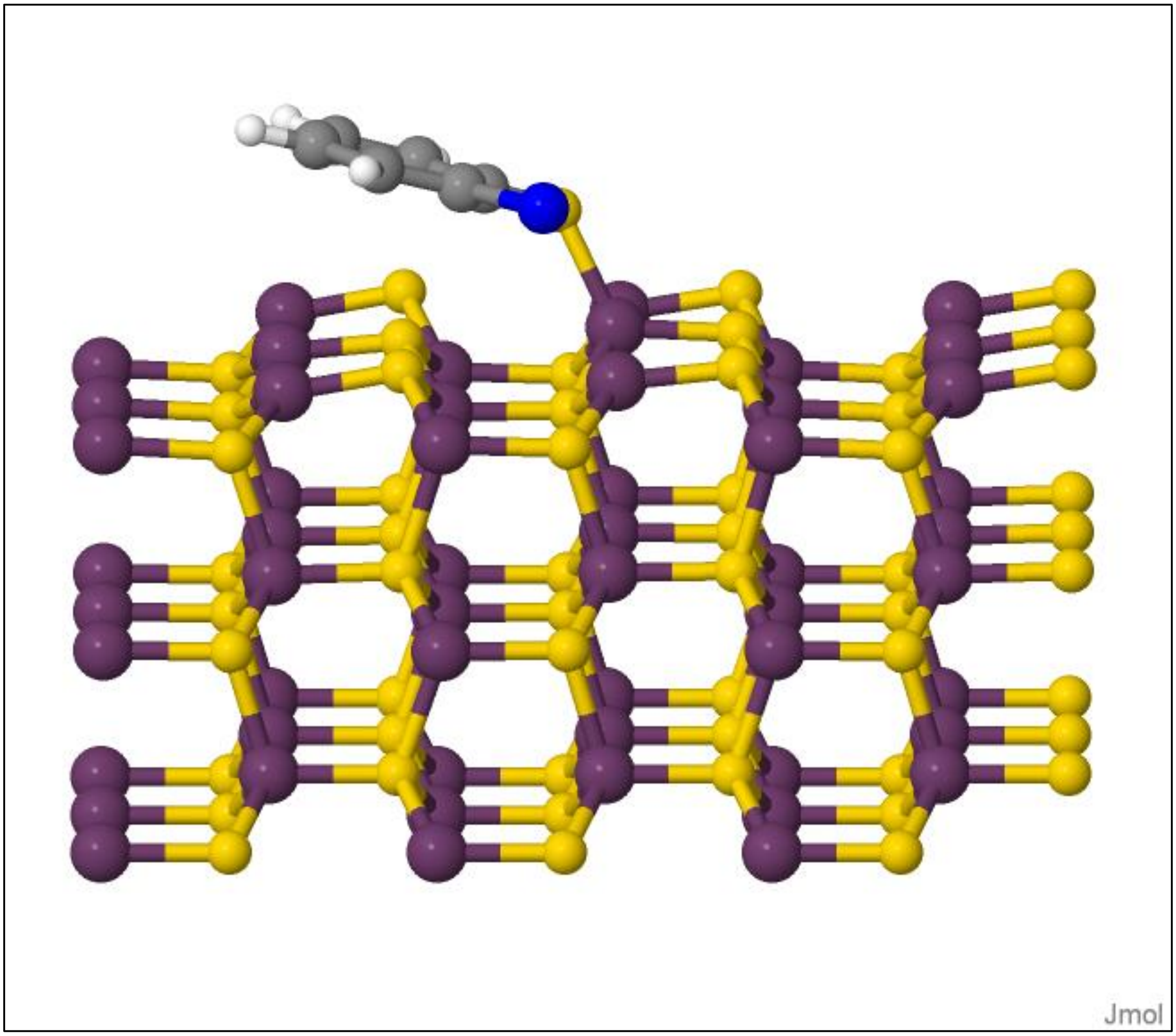

Figure S3. Optimized 3x3x3 ZnO non-polar $(10\bar{1}0)$ model with DCB attached on the surface. Zn atoms are colored purple, O atoms are colored gold, C atoms are colored gray, H atoms are colored white, and Cl atom is colored blue.

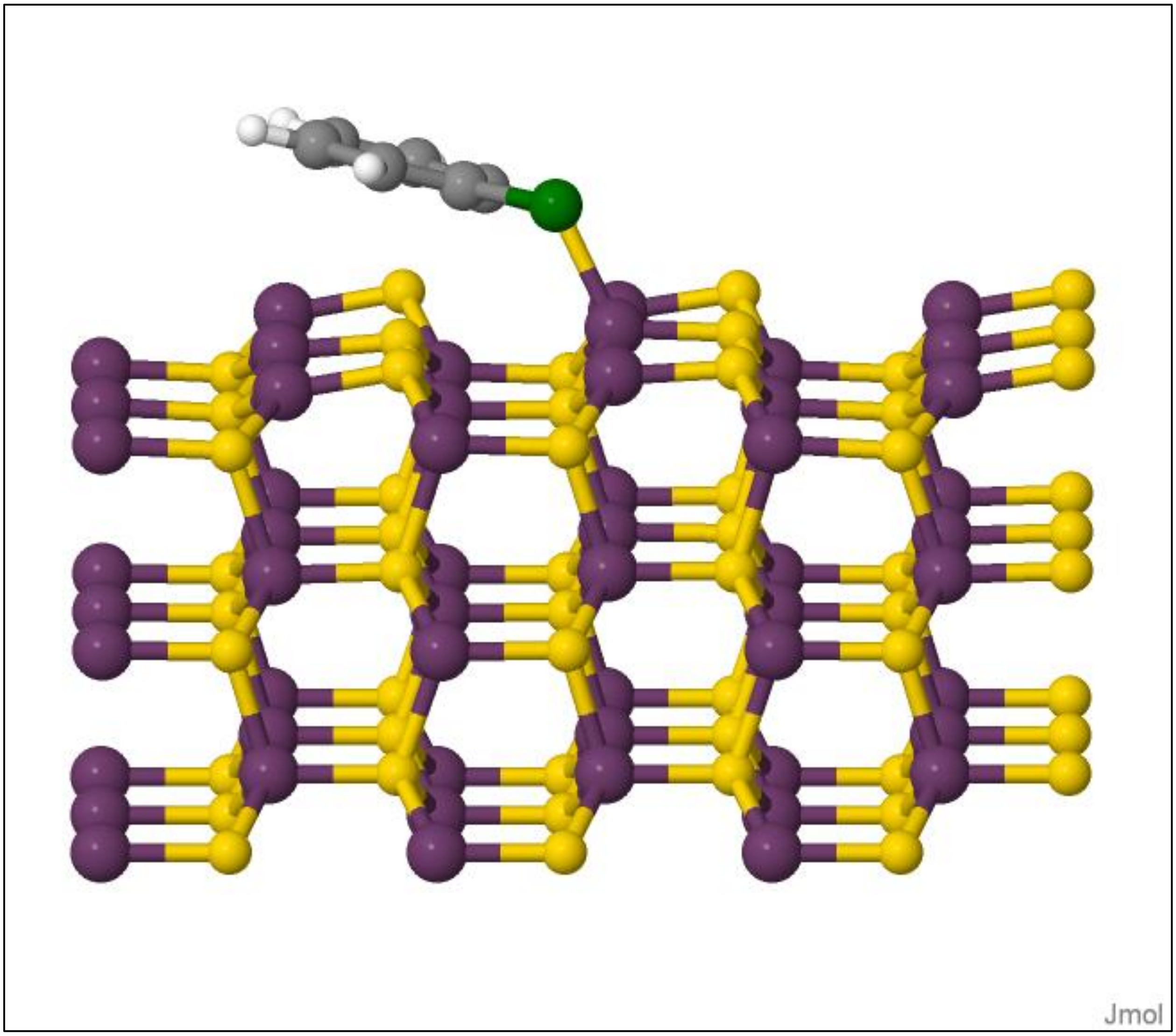

Figure S4. Optimized 3x3x3 ZnO non-polar $(10\bar{1}0)$ model with DBB attached on the surface. Zn atoms are colored purple, O atoms are colored gold, C atoms are colored gray, H atoms are colored white, and Br atom is colored green.

Table S3. Bader charge density analysis of 3x3x3 ZnO non-polar $(10\bar{1}0)$ models

Model	ZnO Surface (e)	Surface hydroxyl or organic (e)	Halogen (X) atom (e)
ZnO-OH	-0.6	-0.4	-
ZnO-DBB	-0.9	0.9	-0.04
ZnO-DCB	-0.9	0.9	-0.20
ZnO-DFB	-0.9	0.9	-0.63

Table S4. Spin Density Analysis of 3x3x3 ZnO non-polar $(10\bar{1}0)$ models

Model	ZnO Surface	Pheno-X	C_6H_4	X	Bridging O
ZnO-DFB	-0.34	-0.55	-0.51	-0.03	-0.18
ZnO-DCB	-0.32	-0.48	-0.45	-0.04	-0.16
ZnO-DBB	-0.33	-0.45	-0.42	-0.02	-0.15
ZnO-OH	-0.49	-	-	-	-0.36

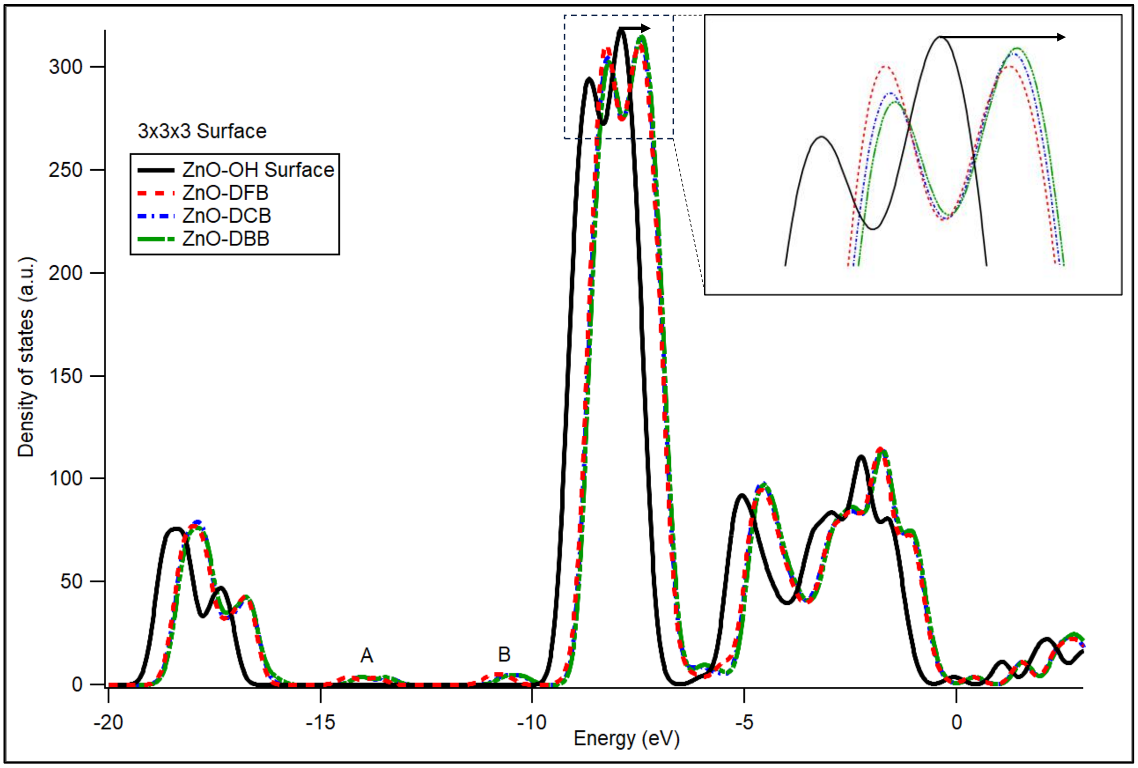

Figure S5. Density of state diagram of ZnO-OH (black line), ZnO-DFB (red line), ZnO-DCB (blue line), and ZnO-DBB (green line) for the 3x3x3 non-polar $(10\bar{1}0)$ models.

List S1. Coordinates in Å of optimized 3x3x2 ZnO non-polar ($10\bar{1}0$) models

OH surface

Zn	7.9949100180	0.8847112962	8.0091969135
Zn	7.9739763652	0.8582836462	2.8136447905
Zn	6.7997765133	3.8932234513	7.9319898567
Zn	6.3633015536	3.6668225570	2.7893103412
Zn	6.6428000471	1.9668693363	-0.0048892082
Zn	6.6625465752	1.9699771600	5.2935961009
Zn	4.7352040408	6.4711063928	2.8160412750
Zn	4.7646357089	6.4672587242	8.0165771023
Zn	5.0393974714	4.7647518723	-0.0023656979
Zn	5.0644233800	4.7745474446	5.2968962605
Zn	4.9006213400	0.9499646956	2.6456182201
Zn	4.9233172636	0.9939017908	7.8621288318
Zn	3.4153111579	7.5969383228	0.0303104683
Zn	3.4301898737	7.5973120587	5.2533682262
Zn	3.2788045384	3.7702932293	2.6514041432
Zn	3.3467835164	3.7656128501	7.8536381553
Zn	3.3057815626	1.9105906371	0.0431882675
Zn	3.3168228271	1.9123346089	5.2479338855
Zn	1.6514327885	6.5772094032	2.6640096279
Zn	1.6604564442	6.5812103181	7.8410208464
Zn	1.6898096484	4.7268327614	0.0344585402
Zn	1.7015787999	4.7176039361	5.2576251770
Zn	1.6246999500	0.9380210040	2.6019001000
Zn	1.6246999500	0.9380210040	7.8056998300
Zn	0.0645934014	7.5311346694	0.0397191395

Zn	0.0659224507	7.5467310919	5.2564146948
Zn	-0.0000000000	3.7520840200	2.6019001000
Zn	-0.0000000000	3.7520840200	7.8056998300
Zn	0.0000000000	1.8760420100	0.0000000000
Zn	0.0000000000	1.8760420100	5.2038002000
Zn	-1.6246999500	6.5661468500	7.8056998300
Zn	-1.6246999500	6.5661468500	2.6019001000
Zn	-1.6246999500	4.6901049600	0.0000000000
Zn	-1.6246999500	4.6901049600	5.2038002000
Zn	-3.2493999000	7.5041680300	0.0000000000
Zn	-3.2493999000	7.5041680300	5.2038002000
O	8.2377301897	1.0006087694	9.8690429105
O	8.2188574222	0.9957065572	4.6612307544
O	6.5829823890	3.7924861800	9.8256850737
O	6.6318336934	3.8126695567	4.6351615880
O	6.5748267153	1.9156363852	1.9990161782
O	6.5660709665	1.9346383122	7.2633212181
O	4.9885265256	6.6097840475	4.6633295185
O	4.9910022580	6.6302980721	9.8738310926
O	4.9552176385	4.7306521159	2.0012169826
O	5.0045845481	4.7131681826	7.2675023889
O	4.9445794775	0.9790790432	4.6313338643
O	4.9226615431	0.9616621272	9.8520802856
O	3.3293819432	7.5448756477	2.0234244187
O	3.3502173434	7.5527993541	7.2397253331
O	3.3346975077	3.7914386206	4.6371140118
O	3.3072639370	3.7830807056	9.8454095286
O	3.2963864076	1.9048840196	2.0272423976

O	3.2903626272	1.8966658499	7.2243616556
O	1.6927053102	6.5993812502	4.6486576029
O	1.6929042856	6.6029758289	9.8328844907
O	1.6733930079	4.7181605149	2.0216453469
O	1.6943280798	4.7045126310	7.2382338258
O	1.6246999500	0.9380210040	4.5902719500
O	1.6246999500	0.9380210040	9.7940721500
O	0.0508218583	7.5250793572	2.0252980697
O	0.0494106950	7.5420950561	7.2365113787
O	-0.0000000000	3.7520840200	4.5902719500
O	-0.0000000000	3.7520840200	9.7940721500
O	0.0000000000	1.8760420100	1.9883719700
O	0.0000000000	1.8760420100	7.1921720500
O	-1.6246999500	6.5661468500	9.7940721500
O	-1.6246999500	6.5661468500	4.5902719500
O	-1.6246999500	4.6901049600	1.9883719700
O	-1.6246999500	4.6901049600	7.1921720500
O	-3.2493999000	7.5041680300	1.9883719700
O	-3.2493999000	7.5041680300	7.1921720500
O	8.2462550016	4.4983938289	6.8380811234
H	7.9114596548	4.4355583927	5.9076337554

DFB surface

Zn	7.9805205408	0.8870676521	8.0310248536
Zn	7.9617681973	0.8579040858	2.8407017006
Zn	6.6761464423	3.8347797044	7.9666638861
Zn	6.3471911175	3.6729729279	2.7762903658
Zn	6.6577380161	1.9857939764	0.0081729368

Zn	6.6290592892	1.9387960092	5.2971035464
Zn	4.7101316408	6.4681278393	2.8185343830
Zn	4.7694847197	6.4858954262	8.0306545395
Zn	5.0406099361	4.7800995132	-0.0005651015
Zn	4.9607765656	4.7909383019	5.2863745807
Zn	4.8977633710	0.9504424779	2.6458825760
Zn	4.9035100220	0.9815546163	7.8724947002
Zn	3.4160517198	7.6094605461	0.0383495930
Zn	3.4263944845	7.6230497240	5.2575010513
Zn	3.2646474821	3.7654652279	2.6448201192
Zn	3.3319617517	3.7710602552	7.8588218170
Zn	3.3065941582	1.9137004338	0.0411681423
Zn	3.3042309165	1.9086295338	5.2493552078
Zn	1.6413657586	6.5784967072	2.6612027032
Zn	1.6649796568	6.5887452824	7.8521144426
Zn	1.6837884663	4.7299245082	0.0329041641
Zn	1.6705404537	4.7337589023	5.2619800186
Zn	1.6246999500	0.9380210040	2.6019001000
Zn	1.6246999500	0.9380210040	7.8056998300
Zn	0.0625664669	7.5367360031	0.0376800469
Zn	0.0559768315	7.5364430391	5.2611958309
Zn	-0.0000000000	3.7520840200	2.6019001000
Zn	-0.0000000000	3.7520840200	7.8056998300
Zn	-0.0000000000	1.8760420100	0.0000000000
Zn	-0.0000000000	1.8760420100	5.2038002000
Zn	-1.6246999500	6.5661468500	7.8056998300
Zn	-1.6246999500	6.5661468500	2.6019001000
Zn	-1.6246999500	4.6901049600	0.0000000000

Zn	-1.6246999500	4.6901049600	5.2038002000
Zn	-3.2493999000	7.5041680300	0.0000000000
Zn	-3.2493999000	7.5041680300	5.2038002000
O	8.2534082720	1.0178021342	9.8831404477
O	8.2551040360	1.0438320987	4.6847085394
O	6.6184294715	3.8278321200	9.8722365304
O	6.4908736770	3.7710654098	4.6471729616
O	6.5824046250	1.9212119371	2.0160502108
O	6.5545271068	1.9389956398	7.2701378682
O	4.9877401169	6.6347701471	4.6629768295
O	5.0018275452	6.6500648684	9.8864465482
O	4.9420877533	4.7389528625	2.0059038305
O	5.0120232668	4.7271257179	7.2654598157
O	4.9344854359	0.9689472210	4.6339095335
O	4.9351531471	0.9742215078	9.8588393899
O	3.3219389799	7.5599958935	2.0358877233
O	3.3564742379	7.5663091604	7.2431799058
O	3.2905722923	3.7902635749	4.6339778253
O	3.3100195318	3.7909053683	9.8457515302
O	3.2996518776	1.9053664175	2.0260517418
O	3.2933506434	1.9081712200	7.2258296140
O	1.7014646467	6.6189503181	4.6489199435
O	1.6979719066	6.6108645134	9.8408135028
O	1.6708276900	4.7245650494	2.0196864304
O	1.6977206511	4.7179983179	7.2408384174
O	1.6246999500	0.9380210040	4.5902719500
O	1.6246999500	0.9380210040	9.7940721500
O	0.0505069259	7.5316379258	2.0231078073

O	0.0552796524	7.5427323888	7.2399932815
O	-0.0000000000	3.7520840200	4.5902719500
O	-0.0000000000	3.7520840200	9.7940721500
O	-0.0000000000	1.8760420100	1.9883719700
O	-0.0000000000	1.8760420100	7.1921720500
O	-1.6246999500	6.5661468500	9.7940721500
O	-1.6246999500	6.5661468500	4.5902719500
O	-1.6246999500	4.6901049600	1.9883719700
O	-1.6246999500	4.6901049600	7.1921720500
O	-3.2493999000	7.5041680300	1.9883719700
O	-3.2493999000	7.5041680300	7.1921720500
C	8.0741728871	6.1305735842	5.5719977960
C	8.5718322927	4.8017236810	5.8591767074
C	9.2950248584	4.1563760975	4.7918131470
C	9.4678301552	4.7819204383	3.5728891473
C	8.9401442544	6.0706502788	3.3440045071
C	8.2402170960	6.7492632854	4.3529373608
H	9.6247140378	3.1316889185	4.9732041287
H	10.0007776488	4.2707197339	2.7704968895
H	9.0746195389	6.5469732552	2.3728433099
H	7.8168693803	7.7403757952	4.1969894042
F	7.4344100561	6.7747266236	6.5734059165
O	8.4162475838	4.2430999964	7.0023362640

DCB surface

Zn	7.9910914558	0.8655484154	8.0466963375
Zn	7.9682532544	0.8433014007	2.8577082888
Zn	6.6965204196	3.8242519180	7.9803928843

Zn	6.3549913831	3.6637747992	2.7864185440
Zn	6.6637046731	1.9729669753	0.0234206385
Zn	6.6389065036	1.9278333317	5.3103610937
Zn	4.7162587006	6.4538743102	2.8370738307
Zn	4.8525746188	6.4928950976	8.0039600533
Zn	5.0490650409	4.7758416104	0.0017246508
Zn	4.9741914922	4.7729696082	5.3055055596
Zn	4.9029941792	0.9451810630	2.6586932202
Zn	4.9165976808	0.9782156305	7.8794541227
Zn	3.4369293958	7.5948856855	0.0438271658
Zn	3.4276867902	7.6071807792	5.2796843095
Zn	3.2749436284	3.7598354375	2.6529513374
Zn	3.3395127249	3.7664298139	7.8725243187
Zn	3.3094339441	1.9059334057	0.0504155972
Zn	3.3100679680	1.9035859850	5.2616596928
Zn	1.6514773214	6.5739335332	2.6668838687
Zn	1.6828989991	6.5794763904	7.8641790469
Zn	1.6888493679	4.7252954894	0.0396401829
Zn	1.6786356541	4.7260180646	5.2696014638
Zn	1.6246999500	0.9380210040	2.6019001000
Zn	1.6246999500	0.9380210040	7.8056998300
Zn	0.0688205999	7.5286503412	0.0457702971
Zn	0.0613944284	7.5283510475	5.2724234553
Zn	-0.0000000000	3.7520840200	2.6019001000
Zn	-0.0000000000	3.7520840200	7.8056998300
Zn	0.0000000000	1.8760420100	0.0000000000
Zn	0.0000000000	1.8760420100	5.2038002000
Zn	-1.6246999500	6.5661468500	7.8056998300

Zn	-1.6246999500	6.5661468500	2.6019001000
Zn	-1.6246999500	4.6901049600	0.0000000000
Zn	-1.6246999500	4.6901049600	5.2038002000
Zn	-3.2493999000	7.5041680300	0.0000000000
Zn	-3.2493999000	7.5041680300	5.2038002000
O	8.2611064343	1.0089421861	9.8986028610
O	8.2603492790	1.0259789595	4.7018169593
O	6.6260208957	3.8140955423	9.8856129207
O	6.4954702621	3.7525980082	4.6568315907
O	6.5897468397	1.9077135132	2.0308800672
O	6.5743999971	1.9296539928	7.2841303334
O	4.9853523036	6.6161483465	4.6803397952
O	5.0166577235	6.6406204225	9.8775403844
O	4.9511789048	4.7304271171	2.0109069953
O	5.0194887772	4.6993965738	7.2808428175
O	4.9379102517	0.9600974016	4.6462566783
O	4.9394853708	0.9667138837	9.8667240026
O	3.3358211296	7.5495859767	2.0427879213
O	3.3793476491	7.5477572097	7.2641598093
O	3.2965065367	3.7821861050	4.6419329137
O	3.3148141762	3.7843485009	9.8587839826
O	3.3050769443	1.8985973298	2.0352412500
O	3.3009050249	1.8997762375	7.2382664027
O	1.7053294330	6.6107184345	4.6573906888
O	1.7064012544	6.6056907975	9.8517030346
O	1.6780066960	4.7179619702	2.0271670814
O	1.7032365956	4.7089273602	7.2483193514
O	1.6246999500	0.9380210040	4.5902719500

O	1.6246999500	0.9380210040	9.7940721500
O	0.0567808228	7.5261120164	2.0318740372
O	0.0676880869	7.5362986623	7.2524391425
O	-0.0000000000	3.7520840200	4.5902719500
O	-0.0000000000	3.7520840200	9.7940721500
O	0.0000000000	1.8760420100	1.9883719700
O	0.0000000000	1.8760420100	7.1921720500
O	-1.6246999500	6.5661468500	9.7940721500
O	-1.6246999500	6.5661468500	4.5902719500
O	-1.6246999500	4.6901049600	1.9883719700
O	-1.6246999500	4.6901049600	7.1921720500
O	-3.2493999000	7.5041680300	1.9883719700
O	-3.2493999000	7.5041680300	7.1921720500
C	8.1365029660	6.2135540860	5.5861970923
C	8.5844896849	4.8690255393	5.8982012051
C	9.2725191686	4.1693459839	4.8392559104
C	9.4372705105	4.7330704517	3.5887722395
C	8.9288011111	6.0173690104	3.3159548330
C	8.2842547103	6.7557860410	4.3211786097
H	9.5917079120	3.1492516447	5.0591864023
H	9.9479499093	4.1746589379	2.8022363060
H	9.0465493897	6.4516367894	2.3223263388
H	7.9040581723	7.7560576940	4.1195919950
Cl	7.4596946886	7.1694233593	6.8685371347
O	8.4238971804	4.3223802426	7.0456695382

DBB surface

Zn	8.0017051452	0.8579928578	8.0042073600

Zn	7.9633757894	0.8382722497	2.8096605522
Zn	6.6913127994	3.8072117704	7.9512893578
Zn	6.3483360454	3.6607938915	2.7367223499
Zn	6.6596329554	1.9607432494	-0.0147636267
Zn	6.6418905467	1.9247882589	5.2667589921
Zn	4.7224058629	6.4586157373	2.8027603696
Zn	4.9176449516	6.5153796913	7.9421194560
Zn	5.0487111405	4.7814379923	-0.0401253987
Zn	4.9905936406	4.7729323199	5.2681933724
Zn	4.8947572782	0.9406832108	2.6342887380
Zn	4.9179855497	0.9724972980	7.8466199394
Zn	3.4448768772	7.5903935932	-0.0009935367
Zn	3.4408471764	7.6094851724	5.2426336427
Zn	3.2752107646	3.7649041622	2.6278032029
Zn	3.3461935745	3.7716616333	7.8434798914
Zn	3.3148019616	1.9094654776	0.0290844482
Zn	3.3131925839	1.9061366899	5.2411881179
Zn	1.6547971026	6.5762874391	2.6422350217
Zn	1.6985618386	6.5781238266	7.8390737829
Zn	1.6936028500	4.7262003757	0.0241718106
Zn	1.6844256807	4.7280140084	5.2508586274
Zn	1.6246999500	0.9380210040	2.6019001000
Zn	1.6246999500	0.9380210040	7.8056998300
Zn	0.0739603226	7.5290669272	0.0252619452
Zn	0.0676706027	7.5256247828	5.2551166946
Zn	-0.0000000000	3.7520840200	2.6019001000
Zn	-0.0000000000	3.7520840200	7.8056998300
Zn	0.0000000000	1.8760420100	0.0000000000

Zn	0.0000000000	1.8760420100	5.2038002000
Zn	-1.6246999500	6.5661468500	7.8056998300
Zn	-1.6246999500	6.5661468500	2.6019001000
Zn	-1.6246999500	4.6901049600	0.0000000000
Zn	-1.6246999500	4.6901049600	5.2038002000
Zn	-3.2493999000	7.5041680300	0.0000000000
Zn	-3.2493999000	7.5041680300	5.2038002000
O	8.2629490660	1.0054610983	9.8582077410
O	8.2655876611	1.0251401054	4.6521728464
O	6.6217467364	3.8049548609	9.8586733023
O	6.5015557691	3.7469639096	4.6071320541
O	6.5784063597	1.8995991442	1.9909557719
O	6.5813114781	1.9174196730	7.2419939088
O	5.0027992856	6.6193730862	4.6456108519
O	5.0239053005	6.6419373229	9.8315209201
O	4.9470341057	4.7355269054	1.9718943205
O	5.0238612332	4.6926362516	7.2451714495
O	4.9368500272	0.9559018069	4.6215380485
O	4.9357237131	0.9631140226	9.8352700979
O	3.3385356310	7.5439874595	2.0012329998
O	3.3942342784	7.5387874431	7.2275002123
O	3.3025560120	3.7845065174	4.6167306913
O	3.3158243985	3.7847108829	9.8325128039
O	3.2995763974	1.9005015600	2.0150676428
O	3.2986587757	1.8977785462	7.2181721558
O	1.7120829857	6.6123948967	4.6314723879
O	1.7099628079	6.6060344721	9.8286711857
O	1.6730115174	4.7178396335	2.0114992292

O	1.6999936378	4.7052263425	7.2300409469
O	1.6246999500	0.9380210040	4.5902719500
O	1.6246999500	0.9380210040	9.7940721500
O	0.0542193062	7.5217083101	2.0115190536
O	0.0704244881	7.5257005342	7.2343108349
O	-0.0000000000	3.7520840200	4.5902719500
O	-0.0000000000	3.7520840200	9.7940721500
O	0.0000000000	1.8760420100	1.9883719700
O	0.0000000000	1.8760420100	7.1921720500
O	-1.6246999500	6.5661468500	9.7940721500
O	-1.6246999500	6.5661468500	4.5902719500
O	-1.6246999500	4.6901049600	1.9883719700
O	-1.6246999500	4.6901049600	7.1921720500
O	-3.2493999000	7.5041680300	1.9883719700
O	-3.2493999000	7.5041680300	7.1921720500
C	8.1589805912	6.2338937663	5.6311691471
C	8.5903708833	4.8846438323	5.9188643665
C	9.2421373174	4.1939665031	4.8317469877
C	9.3787352782	4.7723271487	3.5831421772
C	8.8864288965	6.0659841463	3.3412236645
C	8.2805180144	6.7993975640	4.3768723702
H	9.5535387215	3.1660142462	5.0256578469
H	9.8555441624	4.2143086089	2.7754843368
H	8.9848935798	6.5129850083	2.3514245800
H	7.9065759585	7.8064756810	4.1990935361
Br	7.4755680644	7.2843096986	7.0790356449
O	8.4373654479	4.3130570942	7.0571297564

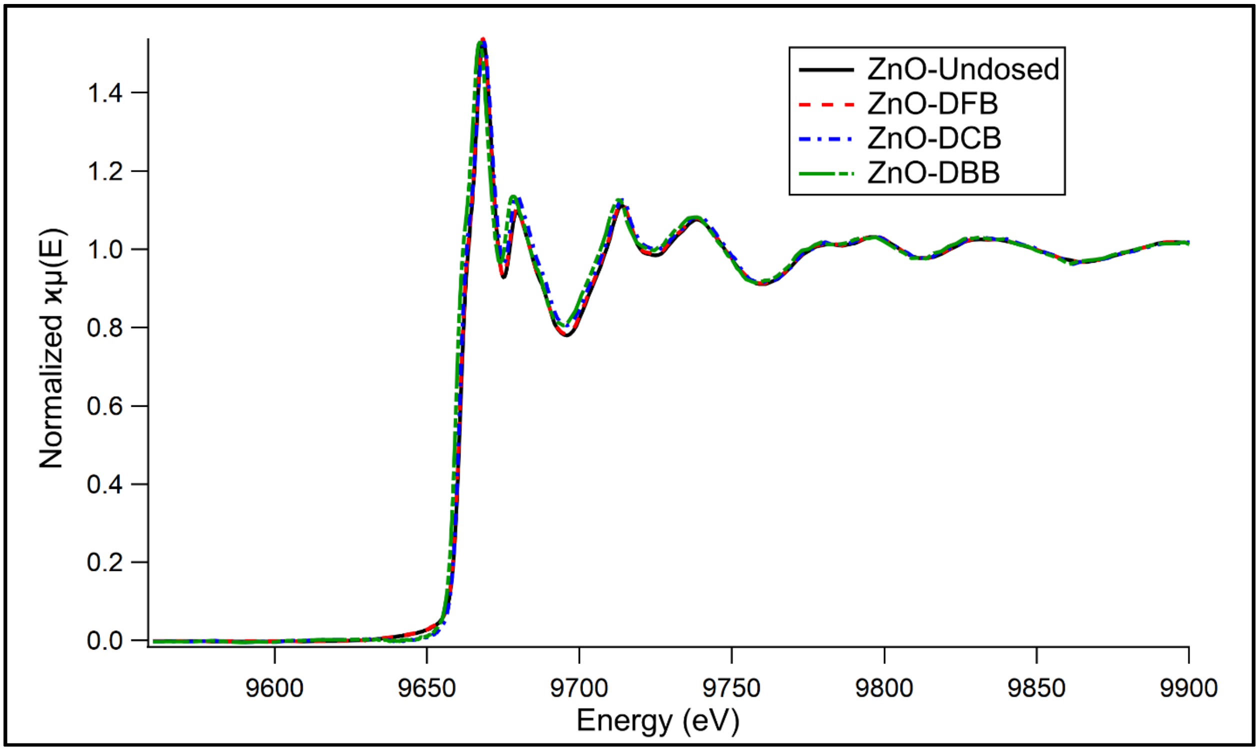

Figure S6. Zn K-edge XANES normalized average spectra of ZnO-Undosed (black line), ZnO-DFB (red line), ZnO-DCB (blue line), and ZnO-DBB (green line).

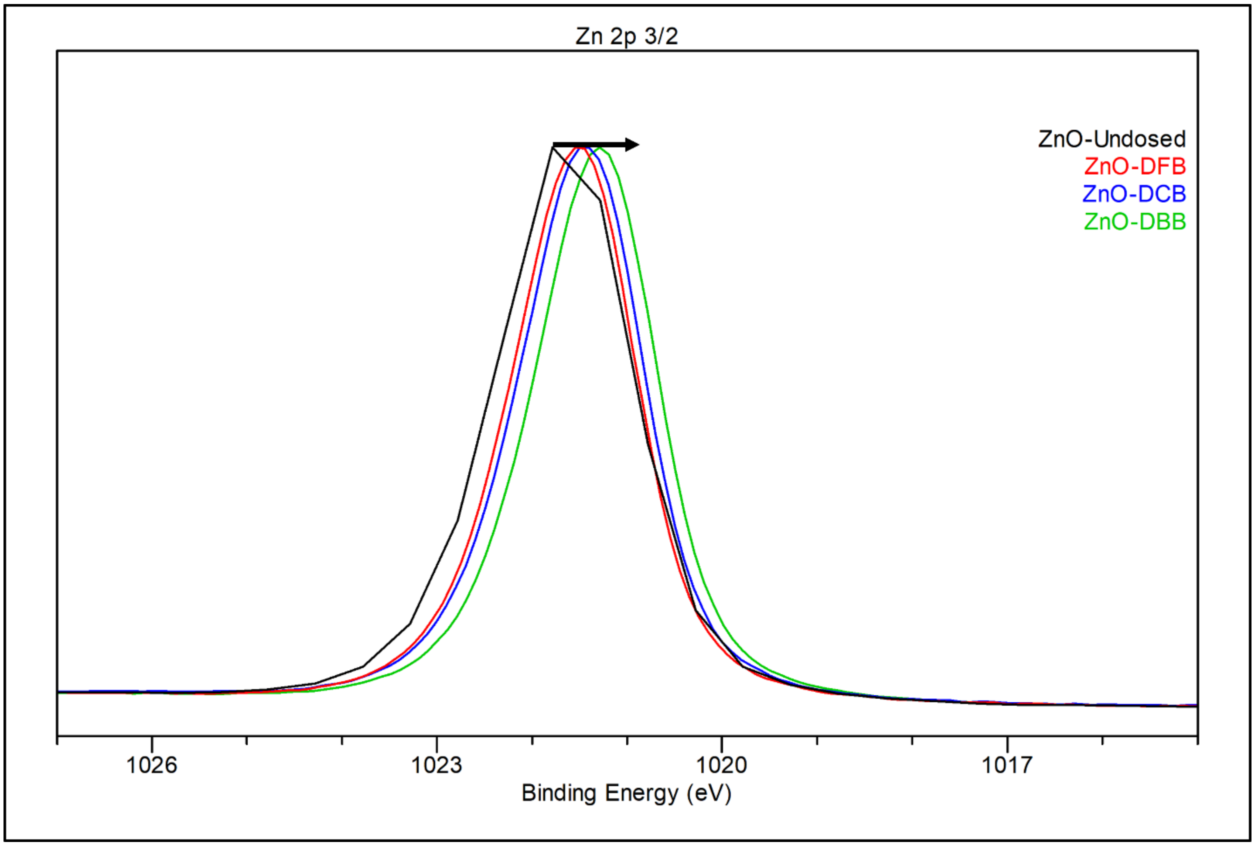

Figure S7. XPS spectra (Zn $2p_{3/2}$ peaks) of ZnO-Undosed (black line), ZnO-DFB (red line), ZnO-DCB (blue line), and ZnO-DBB (green line).
